# Discovery of a Novel Human Pegivirus in Blood Associated with Hepatitis C Virus Co-Infection

**DOI:** 10.1371/journal.ppat.1005325

**Published:** 2015-12-11

**Authors:** Michael G. Berg, Deanna Lee, Kelly Coller, Matthew Frankel, Andrew Aronsohn, Kevin Cheng, Kenn Forberg, Marilee Marcinkus, Samia N. Naccache, George Dawson, Catherine Brennan, Donald M. Jensen, John Hackett, Charles Y. Chiu

**Affiliations:** 1 Abbott Laboratories, Abbott Park, Illinois, United States of America; 2 Department of Laboratory Medicine, University of California, San Francisco, California, United States of America; 3 UCSF-Abbott Viral Diagnostics and Discovery Center, San Francisco, California, United States of America; 4 Center for Liver Diseases, University of Chicago Medical Center, Chicago, Illinois, United States of America; 5 Department of Medicine, Division of Infectious Diseases, University of California, San Francisco, California, United States of America; University of Bonn, GERMANY

## Abstract

Hepatitis C virus (HCV) and human pegivirus (HPgV), formerly GBV-C, are the only known human viruses in the *Hepacivirus* and *Pegivirus* genera, respectively, of the family *Flaviviridae*. We present the discovery of a second pegivirus, provisionally designated human pegivirus 2 (HPgV-2), by next-generation sequencing of plasma from an HCV-infected patient with multiple bloodborne exposures who died from sepsis of unknown etiology. HPgV-2 is highly divergent, situated on a deep phylogenetic branch in a clade that includes rodent and bat pegiviruses, with which it shares <32% amino acid identity. Molecular and serological tools were developed and validated for high-throughput screening of plasma samples, and a panel of 3 independent serological markers strongly correlated antibody responses with viral RNA positivity (99.9% negative predictive value). Discovery of 11 additional RNA-positive samples from a total of 2440 screened (0.45%) revealed 93–94% nucleotide identity between HPgV-2 strains. All 12 HPgV-2 RNA-positive cases were identified in individuals also testing positive for HCV RNA (12 of 983; 1.22%), including 2 samples co-infected with HIV, but HPgV-2 RNA was not detected in non-HCV-infected individuals (p<0.0001), including those singly infected by HIV (p = 0.0075) or HBV (p = 0.0077), nor in volunteer blood donors (p = 0.0082). Nine of the 12 (75%) HPgV-2 RNA positive samples were reactive for antibodies to viral serologic markers, whereas only 28 of 2,429 (1.15%) HPgV-2 RNA negative samples were seropositive. Longitudinal sampling in two individuals revealed that active HPgV-2 infection can persist in blood for at least 7 weeks, despite the presence of virus-specific antibodies. One individual harboring both HPgV-2 and HCV RNA was found to be seronegative for both viruses, suggesting a high likelihood of simultaneous acquisition of HCV and HPgV-2 infection from an acute co-transmission event. Taken together, our results indicate that HPgV-2 is a novel bloodborne infectious virus of humans and likely transmitted via the parenteral route.

## Author Summary

To date, only one human hepacivirus (HCV) and one human pegivirus (HPgV-1/GBV-C) in the family *Flaviviridae* are known to exist. Using unbiased metagenomic next-generation sequencing, we discovered and assembled the genome of a novel pegivirus from plasma corresponding to an HCV-infected patient who died from unknown sepsis. This virus, provisionally named human pegivirus 2 (HPgV-2), is highly divergent, sharing <32% amino acid identity with its nearest relatives, a bat and rodent pegivirus. Identification and sequencing of 11 additional HPgV-2 viruses, revealing 93–94% identity between strains, as well as documented antibody responses using multiple markers, confirm that HPgV-2 is a *bona fide* novel infectious virus of humans. Several lines of evidence, including (1) a documented history of multiple bloodborne exposures in the index patient, (2) parallel detection of HPgV-2 and HCV RNA in an individual during the "window period" between infection and the appearance of detectable antibody, and (3) a tight observed association between HPgV-2 and HCV co-infection, suggest that HPgV-2 is an infectious agent capable of bloodborne transmission.

## Introduction

The only members of the *Hepacivirus* and *Pegivirus* genera that are known to infect humans are Hepatitis C virus (HCV) and human pegivirus (HPgV, formerly GB virus C/GBV-C). HCV infects approximately 3% of the world’s population and can cause liver damage resulting in cirrhosis, hepatocellular carcinoma, and the need for transplantation [1]. In contrast, HPgV infection is considered non-pathogenic, although prevalence can exceed 40% in populations at high risk for exposure to blood-borne agents [2,3]. While controversial, several groups have also reported that co-infection of HPgV and human immunodeficiency virus (HIV) can delay progression to AIDS, presumably by decreasing HIV replication or perturbing the host immune response [3–5]. More recently, co-infection with HPgV in patients with Ebola virus disease has been reported to be associated with improved survival [6].

Novel animal hepaciviruses and pegiviruses have previously been found in multiple animal reservoirs, including bats, rodents, cows, and horses [7–13]. While hepaciviruses such as HCV in humans and GBV-B are established hepatitis agents [14], traditional criteria for classification of viruses as pegiviruses have included phylogenetic relatedness, persistent infection in the host and, importantly, apparent lack of pathogenicity [15,16]. However, the reported discovery of Theiler’s disease-associated virus (EPgV-TDAV) [7], a novel pegivirus associated with acute hepatitis outbreaks in horses, belies this general classification and suggests that at least one member of the *Pegivirus* genus is able to cause hepatitis in its animal host.

Unbiased metagenomic next-generation sequencing (NGS) is an established approach for the detection and discovery of novel pathogens that can be highly divergent and thus undetectable using conventional molecular methods [17]. Here we employed NGS and the sequence-based ultra-rapid pathogen identification (SURPI) bioinformatics pipeline for pathogen detection [18] to identify a novel pegivirus, provisionally named HPgV-2, in an HCV-infected patient who died from sepsis and multi-organ failure of unknown etiology. Subsequent phylogenetic, PCR, and serological analyses confirm that HPgV-2 is a novel blood-borne virus infectious to humans.

## Results

### Discovery and whole-genome sequencing of a novel pegivirus in a hepatitis patient

**Fig 1:**
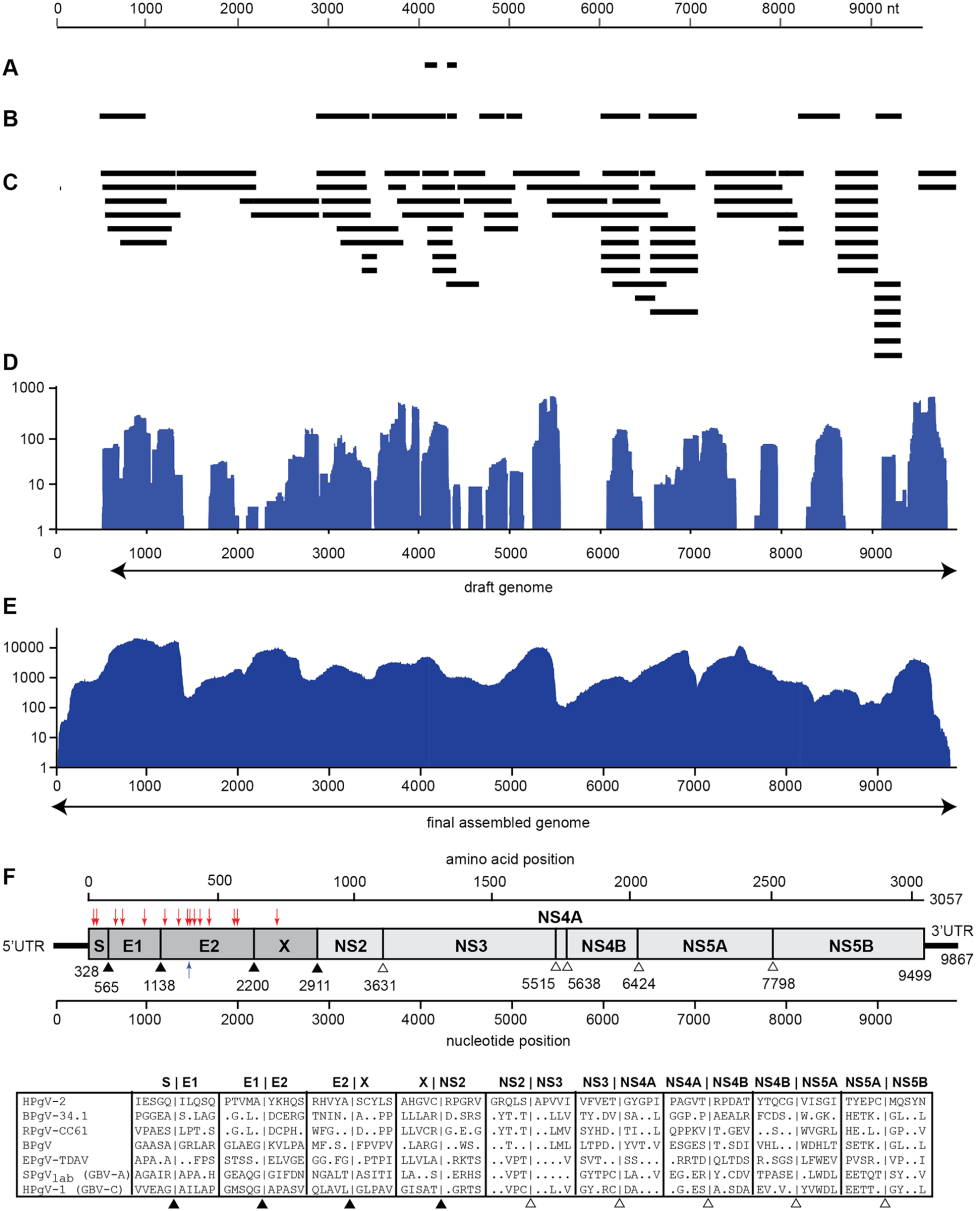
Discovery and whole-genome characterization of HPgV-2. **(A)** Three NGS reads with remote amino acid homology (<60%) to SPgV-A / GBV-A, of which two were overlapping, were detected in a plasma sample from a patient with HCV infection who died of abdominal sepsis (the index case). **(B)** The initial set of contigs generated from *de novo* assembly of HPgV-2 reads that were identified using BLASTx alignment to other pegivirus genomes. **(C)** Gap closure using PCR followed by Sanger sequencing. **(D)** Coverage plot showing mapping of the initial NGS data to the nearly complete (>98%) assembled draft genome. (**E)** Coverage plot showing mapping of the NGS data from a subsequent sequencing run to the complete HPgV-2 genome, after the 5' and 3' ends were recovered using RACE [20]. (**F)** Genomic arrangement of HPgV-2. Putative cleavage sites within the polyprotein are indicated with black triangles (structural proteins) or hollow triangles (non-structural proteins). Arrows denote predicted N-linked (red) or O-linked (blue) glycosylation sites.

Chronic liver disease patients enrolled at the University of Chicago Medical Center (n = 169) were screened by unbiased metagenomic NGS to identify potential novel etiologies. Metagenomic NGS and SURPI analysis done at University of California, San Francisco identified 3 reads in a plasma sample from one patient that were assembled into two contiguous sequences (contigs) sharing 60% amino acid identity to simian pegivirus A (SPgV-A/GBV-A) (Fig 1A). An additional 13.4 million raw metagenomic sequences were then generated from the sample, and 537 reads corresponding to the novel pegivirus (identified by BLASTx translated nucleotide alignment to proteins from SPgV-A, GBV-B, HPgV, and bovine pegivirus / BPgV) were *de novo* assembled using PRICE [19] into 10 contigs spanning >40% of the putative viral genome (Fig 1B). The NGS data were then remapped to these contigs (Fig 1C), followed by PCR and Sanger sequencing to close remaining gaps and recover the nearly complete draft genome (Fig 1D).

The index case, UC0125.US, was a 70 y/o female with a history of sickle-cell disease (SCD), hypertension, HCV infection, and chronic renal insufficiency who was admitted to University of Chicago Medical Center in October of 2011 with abdominal pain. The patient rapidly decompensated after a few days in the hospital and underwent urgent surgical exploration of the abdomen, showing a fulminant colitis secondary to mesenteric ischemia versus gastrointestinal infection. She died approximately 2½ weeks after admission after developing sepsis and resultant multi-organ failure. The autopsy report revealed extensive hemorrhagic necrosis of the abdomen and stage 3 fibrosis of the liver consistent with chronic HCV infection.

The patient worked in the textile industry and had a prior history of alcohol and illegal intravenous drug use (IVDU). She was initially diagnosed with HCV genotype 1a infection in 1998, and failed treatment with interferon-α in 1999, resulting in chronic aminotransferase elevation from 2000–2009, with HCV viral loads ranging from 226,000 to 12,685,000 copies per mL (S1 Fig). A liver biopsy performed in April of 2003 revealed mild portal inflammation and stage 1 fibrosis secondary to chronic HCV infection, and repeat biopsies in 2006 and 2009 showed progression of fibrosis. Notably, the patient had undergone multiple exchange and blood transfusions throughout her lifetime due to repeated vaso-occlusive crises as a result of her SCD. The patient's plasma sample from which pegivirus sequences were originally identified was drawn 8/28/08.

To confirm the presence of a novel pegivirus and exclude the possibility of contamination, a separate aliquot of plasma from the index case collected on the same day (8/28/08) was independently processed at Abbott Laboratories. Metagenomic NGS yielded 249,693 reads out of 16,306,796 (1.53%) mapping to the draft genome. Reads covered 98.4% of the predicted complete genome with an average depth of 3,314X ± 426 reads per nucleotide (Fig 1E). This independently-assembled consensus genome shared 99.73% identity to the draft genome, with every mismatch either conserved or resolving an ambiguous base, and the 5’ end was extended by an additional 306 nucleotides. Due to its structural and phylogenetic similarity to pegiviruses, we provisionally named the novel virus human pegivirus 2 (HPgV-2, strain UC0125.US), in accordance with recently established criteria [16].

### HPgV-2 Genomic Features

Similar to all known pegiviruses, HPgV-2 has a positive-sense RNA genome of length 9,867 nucleotides, with a multi-functional polyprotein encoded by a single open reading frame (ORF) of ~3000 amino acids (aa) and flanked by 5' and 3' untranslated regions (UTRs). The putative UTRs from 11 isolates were nearly identical in sequence (99–100%; S2C Fig), as expected given the constraints of secondary structure. Putative signal peptidase cleavage sites processed by host and viral proteases were predicted by sequence homology with other pegiviruses (Fig 1F). Like other pegiviruses, HPgV-2 was found to have a truncated core protein (S, nucleocapsid), two structural envelope glycoproteins (E1 and E2), an “X” protein of unknown function, and 6 non-structural proteins (NS2, NS3, NS4A, NS4B, NS5A, and NS5B).

At the amino terminus of the polyprotein, six potential initiator (ATG) codons were located in-frame with the predicted ORF. The highest identity of the HPgV-2 5'UTR to rat (RPgV-cc61, 65.9%) and bat (BPgV-34.1, 61.1%) pegivirus sequences, respectively, occurred with the initiator codon positioned at the fifth ATG (nt 326). Beyond this ATG, homology dropped off sharply to 42.8%, suggesting that the beginning of the polyprotein coding sequence was at the fifth ATG and corresponding to a predicted core protein size of 79 aa.

### HPgV-2 Strain Diversity and Phylogeny

**Fig 2:**
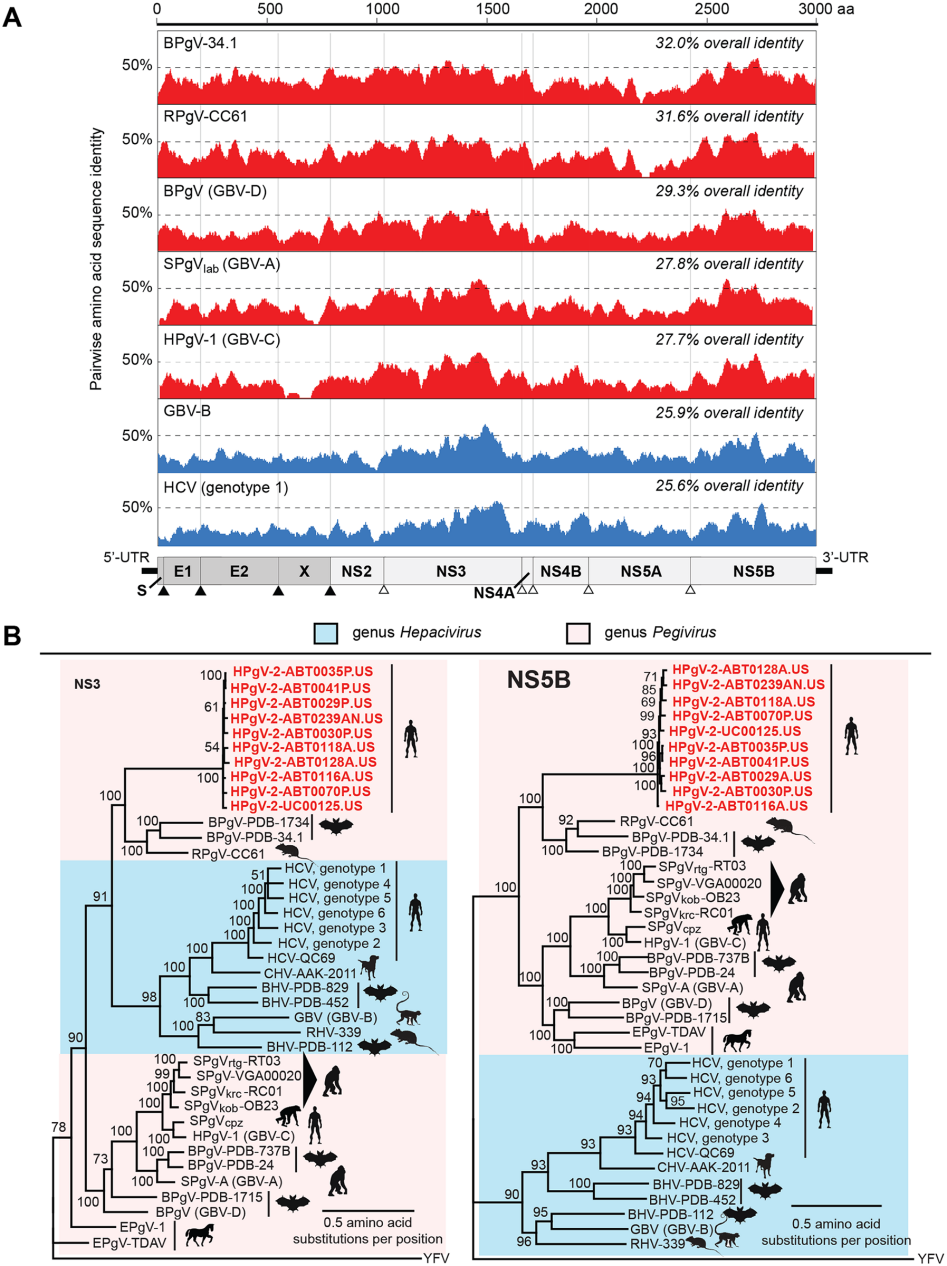
Phylogenetic analysis of HPgV-2 relative to other pegiviruses and hepaciviruses. **(A)** Pairwise amino acid identity plots comparing HPgV-2 (UC0125.US) with other representative pegiviruses (red) and hepaciviruses (blue). The sliding window size is 50 nt. **(B)** Phylogenetic trees of the NS3 (left) and NS5B (right) proteins were constructed for 10 newly sequenced HPgV-2 strains (boldface red), representative hepaciviruses, and all fully sequenced pegiviruses in the NCBI nt database except for members of the simian pegivirus clade, for which 5 representative strains are shown (triangle). Each tree is rooted with yellow fever virus (YFV) as an outgroup.

Comparative alignment of 8 HPgV-2 polyproteins showed 93.8% overall identity at the nucleotide level and 94.7% at the amino acid level,with no apparent hypervariable region observed in the genome. The E1, NS2, NS3, and NS5B genes were the most conserved between strains (92–96% nt identity), whereas more diversity was present in the E2 (91–97%), X (89–94%), NS4A (89–97%), and NS5A (90–94%) proteins. Pairwise identity plots showed that HPgV-2 is substantially divergent from other representative pegiviruses in the National Center for Biotechnology Information (NCBI) nucleotide (nt) database (Fig 2A), including the two most closely related strains, BPgV-34.1 and RPgV-CC61, with which it shared less than 32% overall amino acid identity. Phylogenetic analysis of the NS3, NS5B, and full polyprotein revealed that HPgV-2 strains cluster tightly together on a very deep branch within a clade that includes one rodent and two bat pegiviruses (Fig 2B and S3 Fig).

### Newly developed serologic and molecular diagnostic tools uncover additional strains of HPgV-2

Previous studies have revealed frequent detection of pegivirus GBV-C (HPgV, referred herein as HPgV-1) in HCV-infected individuals [3,15]. Since HPgV-2 was discovered in an HCV co-infected individual (UC0125.US), initial screening of 200 plasmapheresis donors positive for HCV by NAT and Ab testing (HCV NAT+/Ab+) was performed for HPgV-2 using a real-time RT-PCR directed against a region spanning the NS2 and NS3 genes (S2A Fig). Three samples were found to be positive for HPgV-2 RNA (ABT0070P.US, ABT0096P.US, and ABT0188P.US). Metagenomic NGS of these HPgV-2 isolates revealed regions of conservation in the 5’UTR and the NS2-NS3 junction, for which a multiplex assay for detection of HPgV-2 RNA was then developed (S2B Fig). The multiplex assay was also supplemented with primers/probes situated in the 5’UTR of HPgV-1, enabling simultaneous detection of active infection with both HPgV-1 and HPgV-2.

Initial serologic testing of 200 HCV-positive plasmapheresis donors for HPgV-2 was performed using an automated, indirect immunoassay utilizing 16 peptides selected on the basis of predicted HPgV-2 antigenic regions and minimal sequence identity with HCV and HPgV-1 (S4A and S4B Fig). Three of the 16 peptides, located in NS3 (P4), NS5A (P9) and NS4A/B (P16), were predictive of the presence of HPgV-2 RNA and thus were judged to be useful as serological markers (S5 Fig). The remaining 13 peptides were excluded from further testing due to absent or low frequency of reactivity and lack of correlation with RT-PCR. Importantly, the index case was found to be reactive to all three peptides (P4, P9, and P16). A pool of these 3 peptides was used for screening of additional cohorts; samples reactive with the peptide pool were subsequently tested with individual peptides. Reactivity to at least one peptide was considered positive, with reactivity to two or more peptides providing increased confidence (Table 1).

**Table 1:** Serologic and Molecular Detection of HPgV-2

Group	HPgV-2 RNA positive (%)	Antibody reactive to one or more HPgV-2 peptides (%)^a^			Antibody reactive to two or more peptides/ Total Ab+	HPgV-2	HPgV-2	HPgV-1 RNA positive (%)
		Positive (%)	Positive predictive value^d^	Negative predictive value^e^		Ab+/PCR- (cleared)	Ab-/PCR+(acute)	
HCV Ab+/NAT+ (n = 742^b^)	10^c^ (1.3%)	19 (2.6%)	8/19 (42.1%)	721/723 (99.7%)	8/19^f^ (42.2%)	11/732 (1.5%)	2/723 (0.28%)	71/742 (9.5%)
HCV Ab-/NAT+ (n = 240)	1 (0.4%)	3 (1.3%)	0/3 (0%)	236/237 (99.6%)	0/3 (0%)	3/239 (1.3%)	1/237 (0.42%)	NT
HIV (n = 494^b^)	0 (0%)	5 (1.0%)	0/5 (0%)	489/489 (100%)	0/5 (0%)	5/494 (1.0%)	0/489 (0%)	58/494 (11.7%)
HBV (n = 488)	0 (0%)	5 (1.0%)	0/5 (0%)	483/483 (100%)	1/5 (20%)	5/488 (1.0%)	0/483 (0%)	9/488 (1.8%)
Volunteer blood donor (n = 476)	0 (0%)	4 (0.8%)	0/4 (0%)	472/472 (100%)	0/4 (0%)	4/476 (0.8%)	0/472 (0%)	19/452 (4.2%)
Grand total (n = 2440)	11 (0.45%)	36/2440 (1.47%)	8/36 (22.2%)	2401/2404 (99.9%)	9/36 (25%)	28/2429 (1.15%)	3/2404 (0.12%)	157/2176 (7.2%)

Abbreviations: NT, not tested.

^a^Peptides 4, 9, and 16 were utilized as a pool to screen samples.

^b^Two samples were co-infected with HIV and HCV; these two samples were grouped with the HCV Ab+/PCR+ group

^c^Index case (HCV Ab+ /NAT+) has been excluded from totals screened.

^d^Positive predictive value: (# of Ab+/PCR+ samples) ÷ (total # of Ab+ samples) x 100%

^e^Negative predictive value: (# of Ab-/PCR+ samples) ÷ (total # of Ab- samples) x 100%

^f^4 of the 8 were HPgV-2 RNA positive.

We then used the newly developed multiplex RT-PCR and serological assays to screen 2,440 plasma samples from de-identified individuals infected with parentally transmitted viruses (HIV, HBV, or HCV), or volunteer blood donors testing negative for these agents (Table 1). Among 742 HCV NAT+/Ab+ samples, 10 (1.3%) were positive for HPgV-2 RNA, with 8 of these 10 samples seroreactive for HPgV-2. The remaining two seronegative, albeit HPgV-2 RNA positive samples, were from individuals co-infected with HCV and HIV. Among the 19 of 742 (2.6%) HCV NAT+/Ab+ samples seroreactive for HPgV-2, 8 (42.1%) were HPgV-2 RNA positive, indicating that the HPgV-2 serology assay had a positive predictive value (PPV) of 42.1% and negative predictive value (NPV) of 99.7% (721 of 723) for the detection of HPgV-2 RNA (Table 1). Furthermore, 8 of the 19 HPgV-2 Ab+ samples were reactive with two or more peptides, including 4 samples from HPgV-2 RNA positive samples and 4 HPgV-2 RNA negative samples. Screening of 240 HCV-infected individuals prior to seroconversion (HCV NAT+/Ab-) yielded one additional positive, for a total of 11 HPgV-2 RNA positive samples among 2,440 screened, all in patients co-infected with HCV.

Overall, none of the 1,458 non-HCV infected samples were HPgV-2 RNA positive, including samples from 488 HBV NAT+/Ag+ infected individuals, 494 singly-infected HIV NAT+/Ab+ individuals, and 476 volunteer blood donors (Tables 1 and 2). However, in each non-HCV infected group, from 0.8–1.3% of the samples were seroreactive for HPgV-2. Thus, detection of HPgV-2 RNA was statistically different between HCV+ (n = 982) and non-HCV-infected (n = 1458) samples (p value <0.0001 by Fisher’s exact test; Table 2). The frequency of HPgV-2 viremia was also significantly lower than HPgV-1 viremia (p value < 0.0027 by McNemar’s Test) in all of the groups tested (S1 Table).

**Table 2:** Prevalence of HPgV-2 RNA in HCV and non-HCV infected groups.

**Reference Group**			**Comparison Group**			
**Reference Group**	**No. HPgV-2 RNA+ / Total**	**HPgV-2 RNA+ Rate (95% CI)**	**Comparison Group**	**No. HPgV-2 RNA+ / Total**	**HPgV-2 RNA+ Rate (95% CI)**	**P-value by Fisher’s Exact Test** *
HCV Ab+ /NAT+	10/742	1.35% (0.65, 2.46)	HCV Ab-/NAT+	1/240	0.42% (0.01, 2.30)	0.3119
			HIV	0/494	0.0% (0.0, 0.74)	0.0075
			HBV	0/488	0.0% (0.0, 0.75)	0.0077
			Volunteer blood donor	0/476	0.0% (0.0, 0.77)	0.0082
HCV Ab+/ NAT+ and HCV Ab-/NAT+	11/982	1.12% (0.56, 2.00)	Non HCV+ (HIV, HBV, volunteer blood donor)	0/1458	0.0% (0.0, 0.25)	<0.0001
**Reference Group**			**Comparison Group**			
**Reference Group**	**No. HPgV-2 Ab+ / Total**	**HPgV-2 Ab+ Rate (95% CI)**	**Comparison Group**	**No. HPgV-2 Ab+ / Total**	**HPgV-2 Ab+ Rate (95% CI)**	**P-value by Fisher’s Exact Test** *
HCV Ab+/NAT+	19/742	2.56% (1.55, 3.97)	HCV Ab-/NAT+	3/240	1.25% (0.26, 3.61)	0.3181
			HIV	5/494	1.01% (0.33, 2.35)	0.0593
			HBV	5/488	1.02% (0.33, 2.37)	0.0603
			Volunteer blood donor	4/476	0.84% (0.23, 2.14)	0.0319
HCV Ab+/ NAT+ and HCV Ab-/NAT+	22/982	2.24% (1.41 3.37)	Non HCV+ (HIV, HBV, volunteer blood donor)	14/1458	0.96% (0.53, 1.60)	0.0153

*P-value of differences in HPgV-2 RNA (top) and antibody (bottom) prevalence between reference and comparison groups. Index case (HCV Ab+ /NAT+) has been excluded from totals screened.

Notably, all 12 HPgV-2- infected individuals, including the index case, were co-infected with HCV (genotypes 1a, 1b, or 2b), 3 co-infected with HPgV-1 and 2 co-infected with HIV (Table 3). Longitudinal samples were available from two HPgV-2 infected subjects: ABT0030P.US / ABT0033P.US (collected 3 weeks apart) and ABT0035P.US / ABT0041P.US (collected 7 weeks apart). By standard curve analysis, the estimated viral loads for the 12 HPgV-2 strains ranged from 2.5–6.6 log_10_ RNA copies/ml (Table 3). Three HPgV-2 RNA+ samples failed to show reactivity to any of the 3 peptides, suggesting insufficient sensitivity or cases of acute infection prior to seroconversion (Table 1). Using NGS and 5’/3’ RACE (rapid amplification of cDNA ends) [20] across multiple strains, we recovered a total of 8 complete HPgV-2 genomes (including the full 5’UTR and 3’UTR) and 4 partial genomes at 4%−92.3% coverage (Table 3; Fig 2B and 2C).

**Table 3:** Molecular and Serologic Characterization of HPgV-2 Strains

HPgV-2 isolate	HCV			GBV-C RNA	HIV	
	Ab/ RNA	RNA only	Genotype	RNA	RNA	Sub type
UC0125.US	**+**	**-**	1a	**+**	**-**	NA
ABT0096P.US	**+**	**-**	1b	**-**	**-**	NA
ABT0070P.US	**+**	**-**	1b	**-**	**-**	NA
ABT0188P.US	**+**	**-**	1b	**-**	**-**	NA
ABT0055A.US	**+**	**-**	1a	**-**	**-**	NA
ABT0029A.US	**+**	**-**	1a	**+**	**-**	NA
ABT0128A.US	**+**	**-**	1b	**-**	**-**	NA
ABT0239.AN.US	-	**+**	1a	**-**	**-**	NA
ABT0030P.US*	**+**	**-**	1b	**-**	**+**	B
ABT0033P.US*	**+**	**-**	NT	**-**	**+**	NT
ABT0035P.US**	**+**	**-**	2b	**+**	**+**	B
ABT0041P.US**	**+**	**-**	2b	**+**	**+**	B
ABT0116A.US	**+**	**-**	2b	**-**	**-**	NA
ABT0118A.US	**+**	**-**	1b	**-**	**-**	NA

Abbreviations: NA, not applicable; NT, not tested.

*These two samples are from the same individual with bleed dates 26 days apart (sample ABT0030P.US is the earlier sample).

**These two samples are from the same individual with bleed dates 60 days apart (sample ABT0035P.US is the earlier sample).

^†^Italicized numbers show elevated signals, but below the cutoff value.

## Discussion

Here we report the identification of HPgV-2, a second human pegivirus, and recovery of 8 whole and 4 partial genome sequences from 12 strains, all in HCV-infected patients. HPgV-2 was found to be highly divergent, sharing <32% amino acid identity with other pegiviruses. Notably, no cases of active HPgV-2 infection were found in 494 singly HIV-infected individuals, 488 HBV-infected individuals, or 476 volunteer blood donors, as compared to detection of HPgV-2 in 12 of 983 HCV-infected individuals (p<0.0001). The observed overall nucleotide diversity (93.0%-94.4% identity between strains), together with detection of antibodies directed against multiple proteins, establish that HPgV-2 is a *bona fide* infectious agent of humans.

Utilizing the prototype serological assay described herein, 9 of 12 HPgV-2 RNA positive samples were seroreactive, with the majority of these samples (67%; 6 of 9, including the index case) reactive to two or more peptides (Table 3). HPgV-2 seronegative results in three RNA positive individuals may be explained by (1) recent infection, as evidenced by higher viral loads relative to the other HPgV-2 samples (Table 3), (2) host-related factors, such as an immunocompromised state (e.g. HIV infection), resulting in a lack of antibody production, and/or (3) viral amino acid polymorphisms that may impact assay performance (S5 Fig). Interestingly, among the three HPgV-2 RNA+/Ab- samples, one was found in an HCV RNA+/Ab- individual (ABT0239AN.US), suggesting an acute HCV infection occurring within the pre-seroconversion "window" and raising the possibility of a likely HCV/HPgV-2 co-transmission event. The overall prevalence of HPgV-2 across all tested cohorts was 1.64%, with HPgV-2 viremia in 0.45% (11 of 2,440) and HPgV-2 seroreactivity but no viremia in 1.15% (28 of 2,429). We postulate that the HPgV-2 seroreactive yet non-viremic cases may reflect resolved HPgV-2 infections for which individuals have cleared the virus.

HPgV-1, originally designated GBV-C, was discovered in 1995–1996 independently by two groups in sera from patients with non-A, non-B hepatitis [21,22]. Although initially suspected to be a cause of chronic hepatitis, HPgV-1 was later found to be lymphotropic (and not hepatotropic), and to date has not been associated with hepatitis or any other acute clinical illness in humans, despite its capacity for persistent viremia [15]. Nevertheless, there is convincing evidence that at least one pegivirus, EPgV-TDAV, is an etiologic agent of hepatitis in horses [7]. Given co-infection with HCV, we are unable to ascertain whether HPgV-2 played a role in the chronic liver disease of our index patient (UC0125.US), or indeed, whether the virus is hepatotropic. However, longitudinal sampling of one individual in the current study did reveal the capacity of HPgV-2 to produce sustained viremia of at least 7 weeks duration.

Initial serological screening suggests that there are significant differences in the markers and epidemiology of HPgV-2 infection as compared to HPgV-1 infection. First, HPgV-2 antibodies were frequently detected for HPgV-2 viremic samples (9 of 12 cases) in the current study, whereas antibodies are uncommonly detected in actively infected HPgV-1 individuals [2]. Second, unlike the case for HPgV-1, where there is frequent association with HIV infection [23], the only HPgV-2 viremic cases for HIV-infected persons were found among those also co-infected with HCV. Third, HPgV-2 viremia was not as prevalent as HPgV-1 viremia in all of the groups tested (Table 1, S1 Table). We found HPgV-1 in 9.5% of HCV-infected individuals and in 4.2% of volunteer blood donors, comparable with other reports [24–29], while HPgV-2 viremia was 1.1% in HCV-infected individuals (p < 0.0001 versus HPgV-1 viremia) and absent in volunteer donors (p < 0.0001). This difference in prevalence may reflect differences in the mode or efficiency of HPgV-2 transmission relative to HCV and HPgV-1. Given the index patient’s multiple bloodborne exposures, the established association of IVDU with HCV transmission, tight co-detection of HPgV-2 with HCV, and the identification of a likely co-transmission event from an individual dually infected with HPgV-2 and HCV but seronegative for both viruses, it is probable that the mode of transmission for HPgV-2 is also via the parenteral route.

Importantly, all HPgV-2 cases identified in the current study were dually infected with HCV. The association with HCV was statistically significant (p<0.0001), but may not be exclusive given the observed low frequency of HPgV-2 seroreactivity in non-HCV infected individuals. The presumptive clearance of HPgV-2 infection in seroreactive HCV negative individuals also raises the possibility that that HCV co-infection may facilitate persistence of HPgV-2. Interestingly, pegivirus and hepacivirus co-infections have also been reported in horses [30]. Whether HPgV-2 co-infection in humans influences the natural history or course of HCV infection will be of interest, although establishing the pathogenic potential of HPgV-2 is likely to be confounded by its apparent association with HCV. The HCV-positive samples tested to date all originated from the United States; ongoing studies will be needed to determine the global prevalence of HPgV-2 viremia and the full extent of its sequence diversity. Since serology can detect past as well as current infection, future refinement of serological diagnostic assays for HPgV-2 will be critical in establishing the true prevalence of HPgV-2 infection in humans.

Studies to date have suggested that pegivirus evolutionary lineages corresponding to each mammalian host tend to cluster together, consistent with a model of co-divergence of the virus with its host [11,13,31,32]. Our report of at least 12 strains of HPgV-2 infecting humans is consistent this pattern, with each strain sharing ~94% identity to one another. Over 80 novel hepaciviruses and pegviruses have now been reported in bats [13], as well as multiple novel hepaciviruses in rodents [9], cattle [8] and horses [7,10,12], underscoring the extraordinary diversity of these viruses in animal reservoirs. Thus, whether HPgV-2 arose early in the evolutionary history of pegiviruses or is the result of a more recent zoonotic spillover event from a mammalian reservoir remains to be established. Although the long phylogenetic branch lengths corresponding to HPgV-2 would indicate early acquisition of this virus by an ancestral mammalian lineage, the high sequence identity between individual strains (94–96%) as compared to the high diversity of HCV genotypes [33,34] suggests either geographical sampling bias or a recent introduction of HPgV-2 into the human population. Further epidemiological and genomic studies will be needed to pinpoint the precise evolutionary origins of HPgV-2.

## Methods

### Ethics

The case patient was consented for enrollment in a research study approved by the University of Chicago Medical Center Institutional Review Board (IRB), and her plasma sample was analyzed by metagenomic NGS using protocols approved by the UCSF IRB. De-identified samples from cohorts of HBV, HCV and HIV-infected individuals, as well as volunteer blood donors testing negative for these agents, were used for the HPgV-2 screening.

### Identification and genome sequencing of human pegivirus 2 (HPgV-2)

Serum samples were collected from a cohort of 169 patients at University of Chicago Medical Center with chronic hepatitis, most negative for infection by hepatitis viruses A through E. The initial 64 serum samples were pooled, filtered with 0.22 μm filter spin columns (Millipore) and treated with a cocktail of Baseline DNase and Turbo DNase (Ambion) at room temperature for 2.5 hours as previously described [35]. Following nuclease treatment, samples were extracted for viral nucleic acids using the QIAamp Viral RNA Mini Kit (Qiagen). Libraries were prepared for unbiased metagenomic NGS as previously described [36]. The remaining 105 samples were pooled into groups of 6 (50 μl each) and similarly treated with DNase, followed by automated bead-based extraction on the EZ1 instrument using the EZ1 Viral Mini Kit 2.0 (Qiagen). Extracted pools were combined in pairs prior to library preparation and barcode indexing using a modified Illumina TruSeq protocol [36]. Libraries were sequenced as 100 nt paired-end reads on 4 lanes of an Illumina HiSeq2000 instrument, generating a total of 754 million raw reads with an average of 11 million paired-end reads per barcode. Reads were processed using the SURPI bioinformatics pipeline [18]. Briefly, using the SNAP nucleotide aligner, human reads were first computationally subtracted, followed by alignment of remaining reads to all sequences in the comprehensive NCBI nt database. Viral and unmatched reads were then *de novo* assembled, translated in all 6 reading frames, and aligned to all viral protein sequences using RAPSearch [37]. We identified 3 paired-end reads corresponding to a single barcode with remote homology to SPgV-A proteins (Fig 1A). Primers targeting 1 of the 3 reads were used to pinpoint the individual sample harboring HPgV-2.

To recover additional HPgV-2 sequence, additional libraries were prepared from the pool containing the HPgV-2 sample using two independent library methods, TruSeq (Illumina) and ThruPLEX (Rubicon Genomics). NGS libraries were barcoded and sequenced on an Illumina MiSeq, generating 13.4 million raw reads. When aligned to existing pegivirus amino acid sequences in the reference NCBI non-redundant (nr) protein database using BLASTx [38] at an e-value of 10^-5^, 1,080 candidate reads were identified, with 537 of these reads assembling into 9 longer contiguous sequences (contigs) (Fig 1B). Contigs were verified and bridged using PCR and Sanger sequencing (Fig 1C). The final draft assembly, consisting of >98% of the predicted genome, included 604 sequences from HiSeq and 2,571 sequences from MiSeq sequencing (Fig 1D), with Sanger sequencing confirmation at >3X coverage.

### Selection of samples for screening

Retrospective screening was performed on various sets of de-identified samples. Two-hundred samples from first-time plasmapheresis donors, testing positive both by an antibody test for HCV (Abbott Laboratories, Abbott Park IL 60064) and a HCV RNA test (Bayer Versant HCV RNA 3.0 assay) were purchased from ProMedDx (Norton, MA). The majority of samples were obtained from the Midwestern USA. In addition, 742 HCV RNA+/ Ab+ samples and 240 HCV RNA+/Ab- samples were purchased from the American Red Cross (Gaithersburg, MD, USA), while 498 HIV RNA+/Ab+ samples (collected from the East Coast, USA) and 488 HBV DNA+/Ab+ samples (collected from the West Coast, USA) were purchased from ProMedDx. Volunteer blood donor samples pre-screened for HCV, HBV, and HIV were purchased from Gulf Coast, Houston TX and were collected mainly from the Southwestern USA.

### Molecular Screening

For initial molecular detection of RNA positive samples by quantitative real-time reverse transcription PCR (qRT-PCR), a stretch of sequence in the highly conserved NS2-NS3 region of flaviviruses was chosen. Automated RNA extractions and quantitative PCR (qPCR) assays were performed on Abbott *m*2000*sp* and *m*2000*rt* instruments, respectively. Primers were synthesized at Europhins MWG Operon (Huntsville, AL): NS23ExF2, 5’-GAAGATCTGCCACCTGGTTT-3’ NS23ExR2, 5’-AGTGTCGCCTTAAGGAAGGA-3’. The 5’-FAM and 3’-TAMRA labeled probes were synthesized at Applied Biosystems (Foster City, CA): NS23ExASPrb2-, 5’-CCACCGGAGCACTCAGCTGG-3’ (S2A Fig). A positive control RNA from nt 3342–4601 was generated by *in vitro* transcription using the MEGAscript kit (Ambion) according to the manufacturer's instructions. qPCR was performed in 25 μl reactions containing 5 μl of sample RNA using the AgPath-ID One-Step RT-PCR kit (Life Technologies; Carlsbad, CA). PCR cycling conditions were according to manufacturer recommendations, except that 45 cycles of amplification were performed.

Using an alignment of newly identified strains, a multiplex qPCR assay for HPgV-2 was subsequently developed on the *m*2000 system (S2B Fig), which permitted the simultaneous detection of HPgV-1 RNA. Detection of HPgV1 was achieved by targeting the 5’ UTR using FP 5’-TGTTGGCCCTACCGGTGTTA-3’ and RP 5’-CCGTACGTGGGCGTCGTT-3’ and three fluorescently labeled probes (5’-VIC- CTCGTCGTTAAACCGAGCCCGTCA-BHQ1-3’, 5’-VIC-CTCGTCGTTAAACCGAGACCGTCA-BHQ1-3’, 5’-VIC-CACGCCGTTAAACCGAGACCGTTA-BHQ1-3’), adapted from Keys, et al [28]. Detection of HPgV2 relied on amplification of the NS2-3 region (FP 5’-GTGGGACACCTCAACCCTGAAG-3’, RP 5’-GGGAAGACAACACCACGATCTGGC-3’, probe 5’-FAM-CCTGGTTTCCAGCTGAGTGCTCC-BHQ1-3’) and the 5’ UTR (FP 5’-CGCTGATCGTGCAAAGGGATG-3’, RP 5’-GCTCCACGGACGTCACACTGG-3’, probe 5’-Quasar670-GCACCACTCCGTACAGCCTGAT-BHQ2-3’). All primers were synthesized by IDT (Coralville, IA) and probes synthesized at Abbott Molecular (Des Plaines, IL). Cycling conditions were as follows: 50°C, 4 minutes (1X); 75°C, 5 minutes (1X); 60°C, 30 minutes (1X); 91°C, 30 seconds; 58°C, 45 seconds (6X); 91°C, 30 second; 60°C, 45 seconds (+2 sec/cycle)(4X); 91°C, 30 second; 60°C, 45 seconds (+2 sec/cycle)(43X-Read). The rTth DNA polymerase enzyme (Roche) was used for reverse transcription and DNA polymerase activity. To confirm results and exclude the possibility of contamination, qPCR and NGS analyses were repeated from multiple independent extracts, all of which successfully detected sequences corresponding to HPgV-2.

### Design and synthesis of HPgV-2 peptides

Peptides were designed to target amino acid sequences found in the HPgV-2 polyprotein of the index case. Predicted surface exposure (hydrophilicity profile and surface probability) and antigenic index scores were computed using Protean (DNAStar, Madison WI, USA), and amino acid stretches from S, E1, E2, NS3, NS3, NS4A, NS4B, NS5A, and NS5B were chosen to be synthesized as peptides. Hydropathy profiles for each ORF were assessed for areas with potential exposure in aqueous solution (e.g. hydrophilicity). Surface probability was determined using the method of Emini *et al*. [39]. Antigenic index was determined using the Jameson-Wolf algorithm, where surface accessibility of residues is combined with predicted backbone flexibility and secondary structure [40] (S4 Fig). Peptides were generated with an amino terminal biotinylation modification (Genscript).

### Serological screening

Antibody detection with biotinylated peptides was performed on an ARCHITECT instrument (Abbott Laboratories). Human serum or plasma was added to a reaction vessel along with sample diluent buffer (e.g. buffering salts and detergents) containing streptavidin beads (Dynabeads M-270, Life Technologies) and a pool of biotinylated peptides (800 ng/ml each). The sample was incubated for 18 minutes to permit solid phase capture of both the biotinylated peptide and the immune complex (antibody complexed to the peptide). Following incubation, unreacted sample was removed, and a chemiluminescent signal-generating conjugate (mouse anti-human IgG conjugated to acridinium) added to the reaction vessel. After conjugate binding to the immobilized human immunoglobulins and a washing step to remove unreacted material, the microparticles were incubated with a chemiluminescent substrate. The amount of luminescence was then measured in relative light units (RLU) using a bioluminescence imager. Negative controls were prepared either by pooling a series of samples from individuals at low risk for viral infection and testing negative for several common bloodborne viruses (HIV, HBV, and HCV), or by reactions lacking the addition of peptides listed in S4 Fig Seroreactivity was defined using a signal to cutoff (S/CO) ratio of 15, which corresponded to approximately 6 standard deviations from the population mean of volunteer donors testing negative with the peptide pool. Testing on HCV, HIV, HBV and volunteer donor cohorts was performed using a pool of peptides 4, 9, and 16, the three peptides that showed the best correlation with RNA positivity.

### Whole-genome sequencing of multiple strains of HPgV-2

Sequence confirmation of the HPgV-2 genome corresponding to the index case (UC0125.US) and whole-genome recovery of additional strains were performed using NGS. Plasma was clarified of particulate matter by centrifugation at 2650*g* for 5 min at room temperature, and samples were pre-treated with benzonase (500 U/ml; (Sigma)) for 3 hrs at 37°C in 1X benzonase buffer (20 mM Tris-Cl pH 7.5, and 10 mM NaCl, 2 mM MgCl_2_) to degrade free DNA and RNA. For extractions, the QiaAMP Viral Mini (Qiagen), MagMax Viral RNA (Ambion), and Abbott RNA or total nucleic acid sample preps on the *m*2000 instrument were performed according to the manufacturer's instructions, all of which successfully recovered HPgV-2. RNA was concentrated using RNA Clean & Concentrator-5 columns (Zymo Research) and eluted in 7 μl of DNase/RNase-Free water.

Randomly primed cDNA libraries were constructed from 5 μl of RNA using the Ovation RNA-SeqV2 kit (NuGen) according to manufacturer's recommendations. cDNA concentrations were measured on a Qubit Fluorometer using dsDNA BR reagents (Molecular Probes/Life Technologies) and diluted to 0.2 ng/μl. One nanogram (5 μl) was used as input for Nextera XT tagmentation (Illumina) according to manufacturer's instructions, and barcoded libraries were amplified with 16 cycles of PCR. Libraries were quantified on a BioAnalyzer 2200 TapeStation, pooled to a final concentration of 6–12 pM in HT buffer with 1% PhiX loading control, and sequenced on an Illumina MiSeq as 150 nt paired-ends reads using v2 chemistry. To exclude reagent contamination, additional experiments were performed with the SMARTer PCR cDNA synthesis (Clontech), Ovation Single Cell RNA-seq, and Ovation Human Blood RNA-seq library systems, each of which also generated HPgV-2 sequence.

Barcodes were de-multiplexed on the MiSeq instrument and reads were filtered for Q-scores above 30. FASTQ files were imported into CLC Genomics Workbench 8.0 software (CLC bio/Qiagen, Aarhus, Denmark), Illumina paired-end reads 1 and 2 were merged (paired-end distance 100–250 nt), and duplicate reads were removed. Reads were trimmed for quality (limit = 0.05) and ambiguity (2 nt max), with reads below 50 nt discarded; paired-end reads and broken pairs were then aligned to the UC0125.US sequence (Fig 1E). The following alignment settings were applied: mismatch = 2, insertion = 3, deletion = 3, length fraction = 0.7, and similarity fraction = 0.8.

Where required, gaps in NGS sequence were filled using One-Step RT-PCR (Qiagen) and Sanger sequenced on an ABI3130 instrument. Sequences of primers used to characterize the index case and fill in additional genomes are available upon request. Contigs from multiple runs of NGS or Sanger data were assembled in Sequencher 5.2.3 software to generate strain consensus sequences, and the polyprotein open reading frame was confirmed in DNAStar SeqBuilder software. Finishing the 5' and 3' ends of the HPgV-2 was performed using the First Choice RLM RACE kit (Ambion) according to the manufacturer's instruction. Predicted polyprotein cleavage sites were obtained by multiple sequence alignments of the HPgV-2 genome with other fully sequenced pegivirus reference genomes using Geneious v8.0 [41] and by identification of predicted signal peptidase cleavage use using the SignalP 4.0 server [42]. Predicted N-linked and O-linked glycosylation sites in the structural proteins of HPgV-2 were obtained using the online NetNGlyc 1.0 [43] and NetOGlyc 4.0 [44] servers, respectively.

### Pairwise identity plots and phylogenetic analysis

Amino acid pairwise identity plots were generated in Geneious by individual alignment of the polyprotein of HPgV-2 with the polyproteins corresponding to BPgV-34.1 (KC796093), RPgV-CC61 (KC815311), GBV-D (GU566734), GBV-C (NC001710), GBV-A (NC001837), GBV-B (NC001655), and HCV genotype 1 (NC004102) using MAFFT v7.017 at default parameters, followed by display of the percentage identity using a sliding window size of 50 nt.

Phylogenetic trees of NS3, NS5A, or the polyprotein sequences for HPgV-2 strains, other pegiviruses, and representative hepaciviruses were constructed in Geneious 8.0 using the Jukes-Cantor model and neighbor-joining algorithm with 10,000 bootstrap replicates used to calculate branch supports. These tree topologies were then refined using a maximum likelihood Bayesian approach with MrBayes V3.2 software (1,000,000 sample trees, 10% of trees discarded as burn-in, convergence defined at an average standard deviation of <0.01). Each tree was rooted with dengue yellow fever virus (YFV) as the outgroup.

### Data availability

The complete or near-complete genome sequences of 10 HPgV-2 strains have been deposited in NCBI GenBank nt (accession numbers KT427407-KT427414 and KU159664-KU159665). Metagenomic NGS data corresponding to plasma samples from HPgV-2 RNA positive patients have been submitted to the NCBI Sequence Read Archive (accession number SRP066211). NGS reads were filtered for exclusion of human sequences by both BLASTn alignment to all primate sequences in the NCBI nt reference database at an e-value cutoff of 10^-8^ [38] and Bowtie2 high-sensitivity local alignment to the human hg38 reference database [45].

### Accession numbers

Viral strains and GenBank accession numbers used for the generation of the phylogenetic trees, in alphabetical order by the abbreviated name used in the figures, are as follows: BPgV / GBV-D (GU566734), BHV-PDB-112 (KC796077), BPgV-PDB-1715 (KC796088), BPgV-PDB-1734 (KC967087), BPgV-PDB-24 (KC796082), BPgV-PDB-34.1 (KC796093), BHV-PDB-452 (KC796090), BPgV-PDB-737B (KC796081), BHV-PDB-829 (KC796074), CHV-AAK-2011 (JF744991), EPgV-TDAV (KC145265), GBV-B (NC_001655), HCV (genotype 1, NC_004102), HCV (genotype 2, NC_009823), HCV (genotype 3, NC_009824), HCV (genotype 4, NC_009825), HCV (genotype 5, NC_009826), HCV (genotype 6, NC_009827), HCV-QC69 (EF108306), HPgV-1 / GBV-C (NC_001710), RHV-339 (KC815310), RPgV-CC61 (KC815311), SPgV-A / GBV-A (AF023425), SPgV-A / GBV-A (NC_001837), SPgV_cpz_ (AF070476), SPgV_kob_-OB23 (KF234530), SPgV_krc_-RC01 (KF234505), SPgV_krc_-RC06 (KF234499), SPgV_krc_-RC07 (KF234510), PgV_krc_-RC08 (KF234501), SPgV_krc_-RC09 (KF234515), SPgV_krc_-RC13 (KF234523), SPgV_krc_-RC14 (KF234513), SPgV_krc_-RC19 (KF234519), SPgV_krc_-RC26 (KF234522), SSPgV_krc_-RC29 (KF234502), SPgV_krc_-RC31 (KF234507), SPgV_krc_-RC32 (KF234503), SPgV_krc_-RC33 (KF234500), SPgV_krc_-RC34 (KF234509), SPgV_krc_-RC40 (KF234504), SPgV_krc_-RC41 (KF234508), SPgV_krc_-RC43 (KF234521), SPgV_krc_-RC47 (KF234517), SPgV_krc_-RC51 (KF234516), SPgV_krc_-RC52 (KF234511), SPgV_krc_-RC53 (KF234512), SPgV_krc_-RC54 (KF234520), SPgV_krc_-RC58 (KF234506), SPgV_krc_-RC59 (KF234524), SPgV_krc_-RC60 (KF234518), SPgV_krc_-RC61 (KF234514), SPgV_krtg_-RT03 (KF234525), SPgV_krtg_-RT05 (KF234528), SPgV_krtg_-RT06 (KF234526), SPgV_krtg_-RT08 (KF234527), SPgV_krtg_-RT11 (KF234529), SPgV-VGA00020 (KP296858), and YFV (yellow fever virus, NC_002031).

## Supporting Information

S1 Table Detection of HPgV-2 and HPgV-1 RNA. (PDF)

S1 Fig Clinical parameters for the HPgV-2 positive index patient (UC0125.US). The graph shows longitudinal plots of aspartase transaminase (AST), alanine transaminase (ALT), and HCV viral levels from 1995–2011. An arrow indicates the date at which plasma was drawn for NGS analysis (8/25/08).
(PDF)

S2 Fig Genomic locations of primers and probes for detection of HPgV-2 by quantitative PCR (qPCR). **(A)** Multiple sequence nucleotide alignment of HPgV-2 strains in the NS2-NS3 region where primers (black boxes) and probe (red box) were placed for the Ag-Path ID qPCR assay. **(B)** Multiple sequence nucleotide alignment of HPgV-2 strains in the NS2-NS3 and 5'UTR regions where primers (black boxes) and probes (red boxes) were placed for the multiplex qPCR assay.
(PDF)

S3 Fig Phylogenetic analysis of the HPgV-2 polyprotein relative to the polyprotein from other pegiviruses and hepaciviruses. The phylogenetic tree was constructed using 8 HPgV-2 strains, representative hepaciviruses, and all fully sequenced pegiviruses in the NCBI GenBank database, including the simian pegiviruses (triangle). Each tree is rooted with yellow fever virus (YFV) as an outgroup.
(PDF)

S4 Fig Indices for HPgV-2 polyprotein analysis and peptides designed for detection of HgPV-2 specific antibody. **(A)** The HPgV-2 polyprotein was assessed for antigenicity, hydropathy, transmembrane domains, and surface exposure probabilities (rows). Light blue bars indicate the locations of selected peptide targets within the genome. (**B)** Amino acid sequences of peptides and percent homology to HCV and GBV-C.
(PDF)

S5 Fig Alignments of HPgV-2 peptides used in the serological assay. Multiple amino acid sequence alignment of HPgV-2 peptides 4, 9, and 16 sequences are shown. The number refers to the numerical designation for each of the 10 strains (left column). Shown also are signal to noise cut-off (S/CO) values for each tested peptide.
(PDF)

## References

[1] Gower E, Estes C, Blach S, Razavi-Shearer K, Razavi H (2014) Global epidemiology and genotype distribution of the hepatitis C virus infection. J Hepatol 61: S45–57. 10.1016/j.jhep.2014.07.027 25086286

[2] Gutierrez RA, Dawson GJ, Knigge MF, Melvin SL, Heynen CA, et al (1997) Seroprevalence of GB virus C and persistence of RNA and antibody. J Med Virol 53: 167–173. 9334929 10.1002/(sici)1096-9071(199710)53:2<167::aid-jmv10>3.0.co;2-g

[3] Mohr EL, Stapleton JT (2009) GB virus type C interactions with HIV: the role of envelope glycoproteins. J Viral Hepat 16: 757–768. 10.1111/j.1365-2893.2009.01194.x 19758271 PMC3543829

[4] Bhattarai N, Stapleton JT (2012) GB virus C: the good boy virus? Trends Microbiol 20: 124–130. 10.1016/j.tim.2012.01.004 22325031 PMC3477489

[5] Tillmann HL, Manns MP (2001) GB virus-C infection in patients infected with the human immunodeficiency virus. Antiviral Res 52: 83–90. 11672817 10.1016/s0166-3542(01)00172-3

[6] Lauck M, Bailey AL, Andersen KG, Goldberg TL, Sabeti PC, et al (2015) GB virus C coinfections in west African Ebola patients. J Virol 89: 2425–2429. 10.1128/JVI.02752-14 25473056 PMC4338908

[7] Chandriani S, Skewes-Cox P, Zhong W, Ganem DE, Divers TJ, et al (2013) Identification of a previously undescribed divergent virus from the Flaviviridae family in an outbreak of equine serum hepatitis. Proc Natl Acad Sci U S A 110: E1407–1415. 10.1073/pnas.1219217110 23509292 PMC3625295

[8] Corman VM, Grundhoff A, Baechlein C, Fischer N, Gmyl A, et al (2015) Highly divergent hepaciviruses from African cattle. J Virol 89: 5876–5882. 10.1128/JVI.00393-15 25787289 PMC4442428

[9] Drexler JF, Corman VM, Muller MA, Lukashev AN, Gmyl A, et al (2013) Evidence for novel hepaciviruses in rodents. PLoS Pathog 9: e1003438 10.1371/journal.ppat.1003438 23818848 PMC3688547

[10] Kapoor A, Simmonds P, Cullen JM, Scheel TK, Medina JL, et al (2013) Identification of a pegivirus (GB virus-like virus) that infects horses. J Virol 87: 7185–7190. 10.1128/JVI.00324-13 23596285 PMC3676142

[11] Kapoor A, Simmonds P, Scheel TK, Hjelle B, Cullen JM, et al (2013) Identification of rodent homologs of hepatitis C virus and pegiviruses. MBio 4: e00216–00213. 10.1128/mBio.00216-13 23572554 PMC3622934

[12] Lyons S, Kapoor A, Schneider BS, Wolfe ND, Culshaw G, et al (2014) Viraemic frequencies and seroprevalence of non-primate hepacivirus and equine pegiviruses in horses and other mammalian species. J Gen Virol 95: 1701–1711. 10.1099/vir.0.065094-0 24814924

[13] Quan PL, Firth C, Conte JM, Williams SH, Zambrana-Torrelio CM, et al (2013) Bats are a major natural reservoir for hepaciviruses and pegiviruses. Proc Natl Acad Sci U S A 110: 8194–8199. 10.1073/pnas.1303037110 23610427 PMC3657805

[14] Schlauder GG, Pilot-Matias TJ, Gabriel GS, Simons JN, Muerhoff AS, et al (1995) Origin of GB-hepatitis viruses. Lancet 346: 447–448. 10.1016/s0140-6736(95)92821-9 7623597

[15] Chivero ET, Stapleton JT (2015) Tropism of Human Pegivirus (formerly known as GB virus C/Hepatitis G virus)and host immunomodulation: insights into a highly successful viral infection. J Gen Virol. 10.1099/vir.0.000086 PMC5744597 25667328

[16] Stapleton JT, Foung S, Muerhoff AS, Bukh J, Simmonds P (2011) The GB viruses: a review and proposed classification of GBV-A, GBV-C (HGV), and GBV-D in genus Pegivirus within the family Flaviviridae. J Gen Virol 92: 233–246. 10.1099/vir.0.027490-0 21084497 PMC3081076

[17] Chiu CY (2013) Viral pathogen discovery. Curr Opin Microbiol 16: 468–478. 10.1016/j.mib.2013.05.001 23725672 PMC5964995

[18] Naccache SN, Federman S, Veeraraghavan N, Zaharia M, Lee D, et al (2014) A cloud-compatible bioinformatics pipeline for ultrarapid pathogen identification from next-generation sequencing of clinical samples. Genome Res 24: 1180–1192. 10.1101/gr.171934.113 24899342 PMC4079973

[19] Ruby JG, Bellare P, Derisi JL (2013) PRICE: software for the targeted assembly of components of (Meta) genomic sequence data. G3 (Bethesda) 3: 865–880. 23550143 10.1534/g3.113.005967 PMC3656733

[20] Yeku O, Frohman MA (2011) Rapid amplification of cDNA ends (RACE). Methods Mol Biol 703: 107–122. 10.1007/978-1-59745-248-9_8 21125486

[21] Linnen J, Wages J Jr., Zhang-Keck ZY, Fry KE, Krawczynski KZ, et al (1996) Molecular cloning and disease association of hepatitis G virus: a transfusion-transmissible agent. Science 271: 505–508. 8560265 10.1126/science.271.5248.505

[22] Simons JN, Leary TP, Dawson GJ, Pilot-Matias TJ, Muerhoff AS, et al (1995) Isolation of novel virus-like sequences associated with human hepatitis. Nat Med 1: 564–569. 7585124 10.1038/nm0695-564

[23] Giret MT, Kallas EG (2012) GBV-C: state of the art and future prospects. Curr HIV/AIDS Rep 9: 26–33. 10.1007/s11904-011-0109-1 22246585

[24] Alvarado-Mora MV, Botelho L, Nishiya A, Neto RA, Gomes-Gouvea MS, et al (2011) Frequency and genotypic distribution of GB virus C (GBV-C) among Colombian population with Hepatitis B (HBV) or Hepatitis C (HCV) infection. Virol J 8: 345 10.1186/1743-422X-8-345 21745373 PMC3142244

[25] Boodram B, Hershow RC, Klinzman D, Stapleton JT (2011) GB virus C infection among young, HIV-negative injection drug users with and without hepatitis C virus infection. J Viral Hepat 18: e153–159. 10.1111/j.1365-2893.2010.01350.x 20738773 PMC3543827

[26] Claret G, Noguera A, Gonzalez-Cuevas A, Garcia-Garcia JJ, Fortuny C, et al (2008) The prevalence of GB virus C/hepatitis G virus RNA among healthy and HCV-infected Catalan children. Eur J Pediatr 167: 991–994. 17965880 10.1007/s00431-007-0624-7

[27] Hofer H, Aydin I, Neumueller-Guber S, Mueller C, Scherzer TM, et al (2011) Prevalence and clinical significance of GB virus type C/hepatitis G virus coinfection in patients with chronic hepatitis C undergoing antiviral therapy. J Viral Hepat 18: 513–517. 10.1111/j.1365-2893.2010.01340.x 20565572

[28] Keys JR, Leone PA, Eron JJ, Alexander K, Brinson M, et al (2014) Large scale screening of human sera for HCV RNA and GBV-C RNA. J Med Virol 86: 473–477. 10.1002/jmv.23829 24178362 PMC3947266

[29] Pawlotsky JM, Roudot-Thoraval F, Muerhoff AS, Pellerin M, Germanidis G, et al (1998) GB virus C (GBV-C) infection in patients with chronic hepatitis C. Influence on liver disease and on hepatitis virus behaviour: effect of interferon alfa therapy. J Med Virol 54: 26–37. 9443106 10.1002/(sici)1096-9071(199801)54:1<26::aid-jmv5>3.0.co;2-r

[30] Ramsay JD, Evanoff R, Wilkinson TE Jr., Divers TJ, Knowles DP, et al (2015) Experimental transmission of equine hepacivirus in horses as a model for hepatitis C virus. Hepatology 61: 1533–1546. 10.1002/hep.27689 25580897

[31] Sibley SD, Lauck M, Bailey AL, Hyeroba D, Tumukunde A, et al (2014) Discovery and characterization of distinct simian pegiviruses in three wild African Old World monkey species. PLoS One 9: e98569 10.1371/journal.pone.0098569 24918769 PMC4053331

[32] Simmonds P, Domingo E (2011) Virus evolution. Curr Opin Virol 1: 410–412. 10.1016/j.coviro.2011.10.021 22440843 PMC7172365

[33] Simmonds P, Holmes EC, Cha TA, Chan SW, McOmish F, et al (1993) Classification of hepatitis C virus into six major genotypes and a series of subtypes by phylogenetic analysis of the NS-5 region. J Gen Virol 74 (Pt 11): 2391–2399. 8245854 10.1099/0022-1317-74-11-2391

[34] Simmonds P (2004) Genetic diversity and evolution of hepatitis C virus—15 years on. J Gen Virol 85: 3173–3188. 15483230 10.1099/vir.0.80401-0

[35] Swei A, Russell BJ, Naccache SN, Kabre B, Veeraraghavan N, et al (2013) The genome sequence of Lone Star virus, a highly divergent bunyavirus found in the Amblyomma americanum tick. PLoS One 8: e62083 10.1371/journal.pone.0062083 23637969 PMC3639253

[36] Grard G, Fair JN, Lee D, Slikas E, Steffen I, et al (2012) A novel rhabdovirus associated with acute hemorrhagic fever in central Africa. PLoS Pathog 8: e1002924 10.1371/journal.ppat.1002924 23028323 PMC3460624

[37] Zhao Y, Tang H, Ye Y (2012) RAPSearch2: a fast and memory-efficient protein similarity search tool for next-generation sequencing data. Bioinformatics 28: 125–126. 10.1093/bioinformatics/btr595 22039206 PMC3244761

[38] Altschul SF, Gish W, Miller W, Myers EW, Lipman DJ (1990) Basic local alignment search tool. J Mol Biol 215: 403–410. 2231712 10.1016/S0022-2836(05)80360-2

[39] Emini EA, Hughes JV, Perlow DS, Boger J (1985) Induction of hepatitis A virus-neutralizing antibody by a virus-specific synthetic peptide. J Virol 55: 836–839. 2991600 10.1128/jvi.55.3.836-839.1985 PMC255070

[40] Jameson BA, Wolf H (1988) The antigenic index: a novel algorithm for predicting antigenic determinants. Comput Appl Biosci 4: 181–186. 2454713 10.1093/bioinformatics/4.1.181

[41] Kearse M, Moir R, Wilson A, Stones-Havas S, Cheung M, et al (2012) Geneious Basic: an integrated and extendable desktop software platform for the organization and analysis of sequence data. Bioinformatics 28: 1647–1649. 10.1093/bioinformatics/bts199 22543367 PMC3371832

[42] Petersen TN, Brunak S, von Heijne G, Nielsen H (2011) SignalP 4.0: discriminating signal peptides from transmembrane regions. Nat Methods 8: 785–786. 10.1038/nmeth.1701 21959131

[43] Gupta R, Jung E, Brunak S (2015) NetNGlyc 1.0 Server.

[44] Steentoft C, Vakhrushev SY, Joshi HJ, Kong Y, Vester-Christensen MB, et al (2013) Precision mapping of the human O-GalNAc glycoproteome through SimpleCell technology. EMBO J 32: 1478–1488. 10.1038/emboj.2013.79 23584533 PMC3655468

[45] Langmead B, Salzberg SL (2012) Fast gapped-read alignment with Bowtie 2. Nat Methods 9: 357–359. 10.1038/nmeth.1923 22388286 PMC3322381

[46] Kapoor A, Kumar A, Simmonds P, Bhuva N, Singh Chauhan L, et al (2015) Virome Analysis of Transfusion Recipients Reveals a Novel Human Virus That Shares Genomic Features with Hepaciviruses and Pegiviruses. MBio 6. 10.1128/mBio.01466-15 PMC4600124 26396247

