# Correction: Discovery of a Novel Human Pegivirus in Blood Associated with Hepatitis C Virus Co-Infection

**DOI:** 10.1371/journal.ppat.1005386

**Published:** 2016-01-06

**Authors:** The PLOS Pathogens Staff

There is an error in Table 3. The columns to the right of the HIV heading are missing. Please see the full version of the table here, which includes the Peptide reactivity (S/CO = 15), HPgV-2 Viral load, and NGS coverage columns.

**Table 3:** Molecular and Serologic Characterization of HPgV-2 Strains

HPgV-2 isolate	HCV			GBV-C RNA	HIV		Peptide reactivity (S/CO = 15)			HPgV-2 Viral load	NGS coverage
	Ab/ RNA	RNA only	Genotype	RNA	RNA	Subtype	4	9	16	Log RNA copies/ml	Percent genome
UC0125.US	**+**	**-**	1a	**+**	**-**	NA	0.9^†^	6.5	13	6.2	99.8
ABT0096P.US	**+**	**-**	1b	**-**	**-**	NA	2.3	6	13.8	3.5	58
ABT0070P.US	**+**	**-**	1b	**-**	**-**	NA	**-**	3.5	*0*.*7*	5.2	100
ABT0188P.US	**+**	**-**	1b	**-**	**-**	NA	3.5	**-**	**-**	2.5	4
ABT0055A.US	**+**	**-**	1a	**-**	**-**	NA	**-**	-	1	3.8	78.3
ABT0029A.US	**+**	**-**	1a	**+**	**-**	NA	**-**	*0*.*8*	1.9	4.6	100
ABT0128A.US	**+**	**-**	1b	**-**	**-**	NA	**-**	4.6	1.6	4.5	92.3
ABT0239.AN.US	-	**+**	1a	**-**	**-**	NA	**-**	-	**-**	5.8	100
ABT0030P.US*	**+**	**-**	1b	**-**	**+**	B	**-**	-	**-**	6.6	99.9
ABT0033P.US*	**+**	**-**	NT	**-**	**+**	NT	**-**	-	**-**	6	4.1
ABT0035P.US**	**+**	**-**	2b	**+**	**+**	B	**-**	-	**-**	5.9	98.5
ABT0041P.US**	**+**	**-**	2b	**+**	**+**	B	**-**	-	**-**	6.2	99.7
ABT0116A.US	**+**	**-**	2b	**-**	**-**	NA	**-**	25.5	26.7	5.2	96.3
ABT0118A.US	**+**	**-**	1b	**-**	**-**	NA	**-**	-	24.8	6.2	99.8

Abbreviations: NA, not applicable; NT, not tested.

*These two samples are from the same individual with bleed dates 26 days apart (sample ABT0030P.US is the earlier sample).

**These two samples are from the same individual with bleed dates 60 days apart (sample ABT0035P.US is the earlier sample).

^†^Italicized numbers show elevated signals, but below the cutoff value.
