# Supplementary material for: Discovery of a Novel Human Pegivirus in Blood Associated with Hepatitis C Virus Co-Infection
Source: PLoS Pathog. 2015 Dec 11;11(12):e1005325. doi: 10.1371/journal.ppat.1005325 (PMC4676677; doi:10.1371/journal.ppat.1005325)
Supplement: S1 Table — (PDF) [file ppat.1005325.s001.pdf]

S1 Table. Detection of HPgV-2 and HPgV-1 RNA

Group	HPgV-2 RNA positive (%)	HPgV-1 RNA positive (%)	P-values by McNemar's test
HCV Ab+/NAT+ (n=742*)	10 / 742 (1.5%)	71 / 742 (9.5%)	<0.0001
HCV Ab-/NAT+ (n=240)	1 / 240 (0.4%)	NT	NA
HIV (n=494)*	1 / 240 (0.4%)	58 / 475 (12.2%)	<0.0001
HBV (n=488)	0 / 488 (0%)	9 / 488 (1.8%)	0.0027
volunteer blood donors (n=476)**	0 / 476 (0%)	19 / 452 (4.2%)	<0.0001

Abbreviations: NT, not tested; NA, not applicable.

* Two samples were co-infected with HIV and HCV: these two samples were grouped with the HCV Ab+ / PCR+ group

** All volunteer donor samples tested negative for HBV, HCV, and HIV.
