# Supplementary figures and images for: Discovery of a Novel Human Pegivirus in Blood Associated with Hepatitis C Virus Co-Infection
Source: PLoS Pathog. 2015 Dec 11;11(12):e1005325. doi: 10.1371/journal.ppat.1005325 (PMC4676677; doi:10.1371/journal.ppat.1005325)

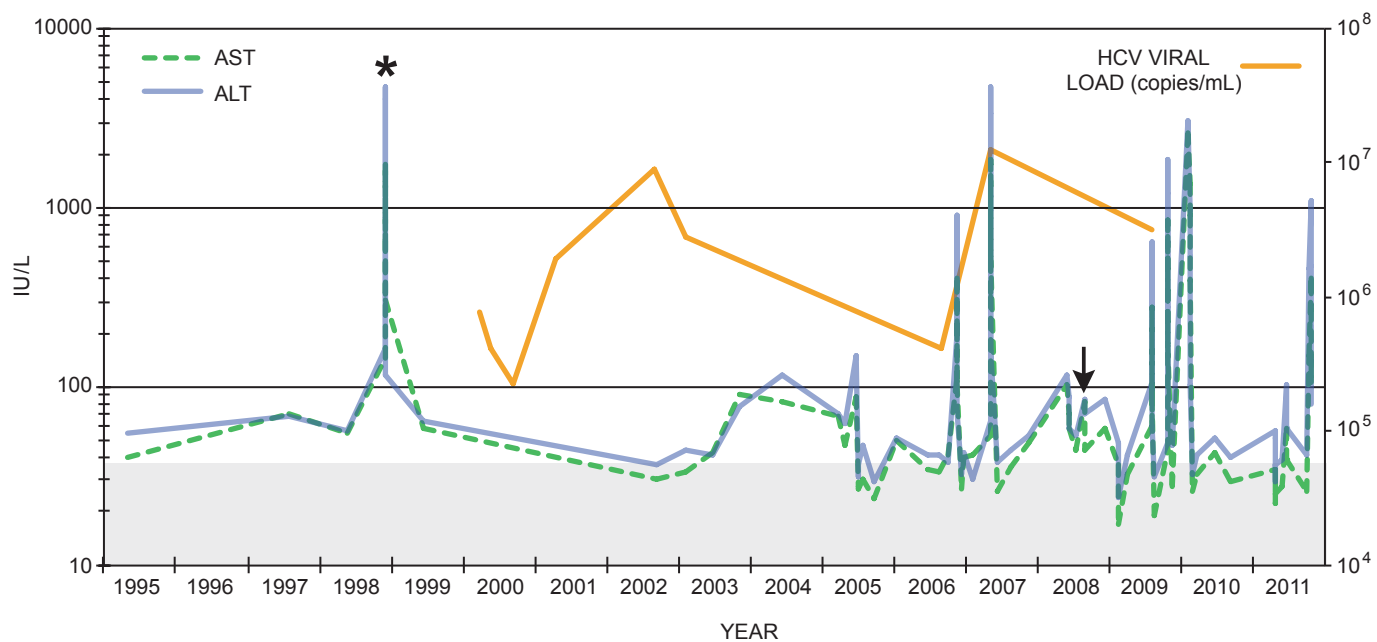

**S1 Figure. Clinical parameters for the HPgV-2 positive index patient (UC0125.US).**

Supplement: S1 Fig — The graph shows longitudinal plots of aspartase transaminase (AST), alanine transaminase (ALT), and HCV viral levels from 1995–2011. An arrow indicates the date at which plasma was drawn for NGS analysis (8/25/08). (PDF) [file ppat.1005325.s002.pdf]
