# Supplementary material for: Discovery of a Novel Human Pegivirus in Blood Associated with Hepatitis C Virus Co-Infection
Source: PLoS Pathog. 2015 Dec 11;11(12):e1005325. doi: 10.1371/journal.ppat.1005325 (PMC4676677; doi:10.1371/journal.ppat.1005325)
Supplement: S2 Fig — (A) Multiple sequence nucleotide alignment of HPgV-2 strains in the NS2-NS3 region where primers (black boxes) and probe (red box) were placed for the Ag-Path ID qPCR assay. (B) Multiple sequence nucleotide alignment of HPgV-2 strains in the NS2-NS3 and 5'UTR regions where primers (black boxes) and probes (red boxes) were placed for the multiplex qPCR assay. (PDF) [file ppat.1005325.s003.pdf]

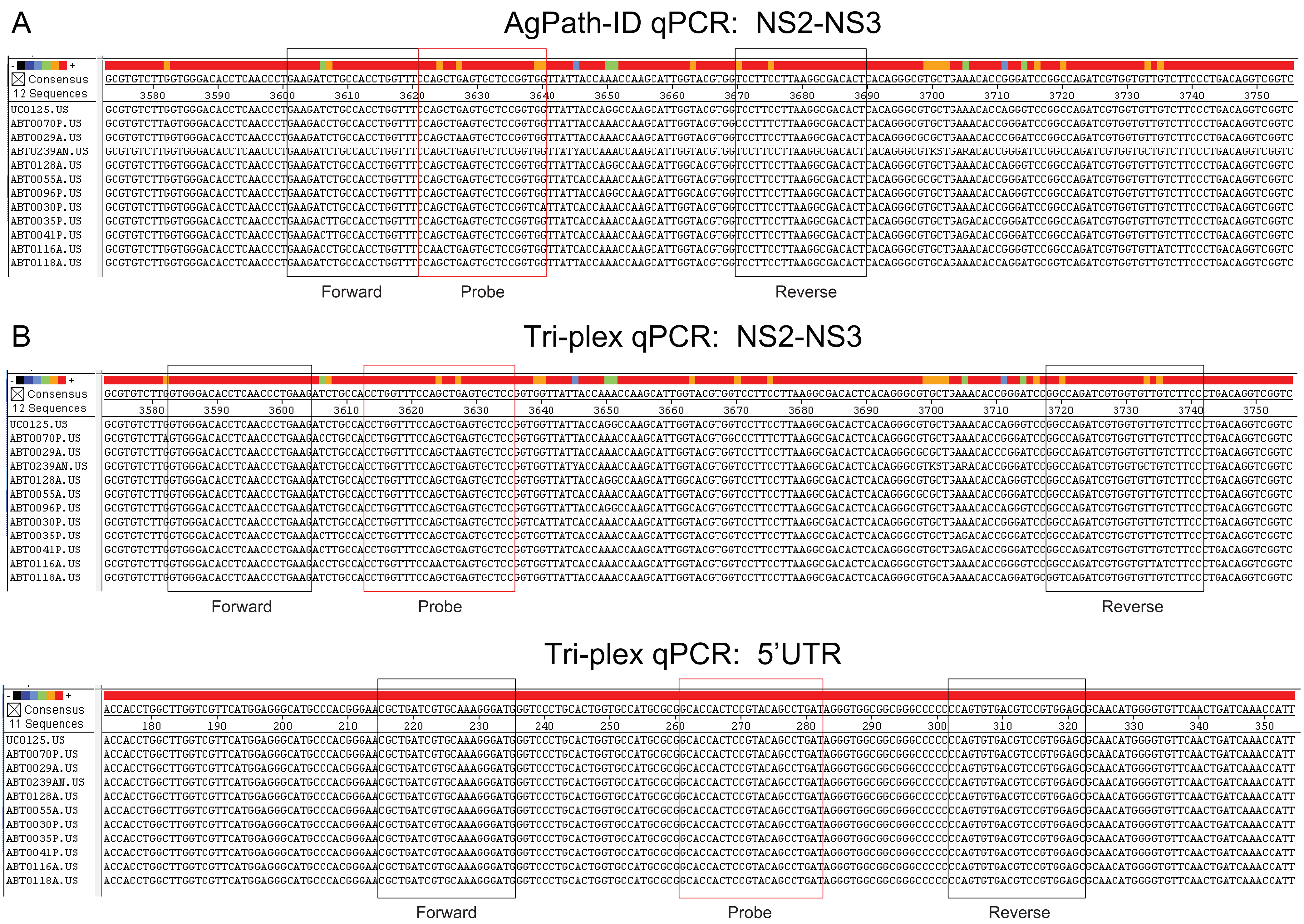


**S2 Figure. Genomic locations of primers and probes for detection of HPgV-2 by quantitative PCR (qPCR)**
