# Supplementary material for: Discovery of a Novel Human Pegivirus in Blood Associated with Hepatitis C Virus Co-Infection
Source: PLoS Pathog. 2015 Dec 11;11(12):e1005325. doi: 10.1371/journal.ppat.1005325 (PMC4676677; doi:10.1371/journal.ppat.1005325)
Supplement: S4 Fig — (A) The HPgV-2 polyprotein was assessed for antigenicity, hydropathy, transmembrane domains, and surface exposure probabilities (rows). Light blue bars indicate the locations of selected peptide targets within the genome. (B) Amino acid sequences of peptides and percent homology to HCV and GBV-C. (PDF) [file ppat.1005325.s005.pdf]

A

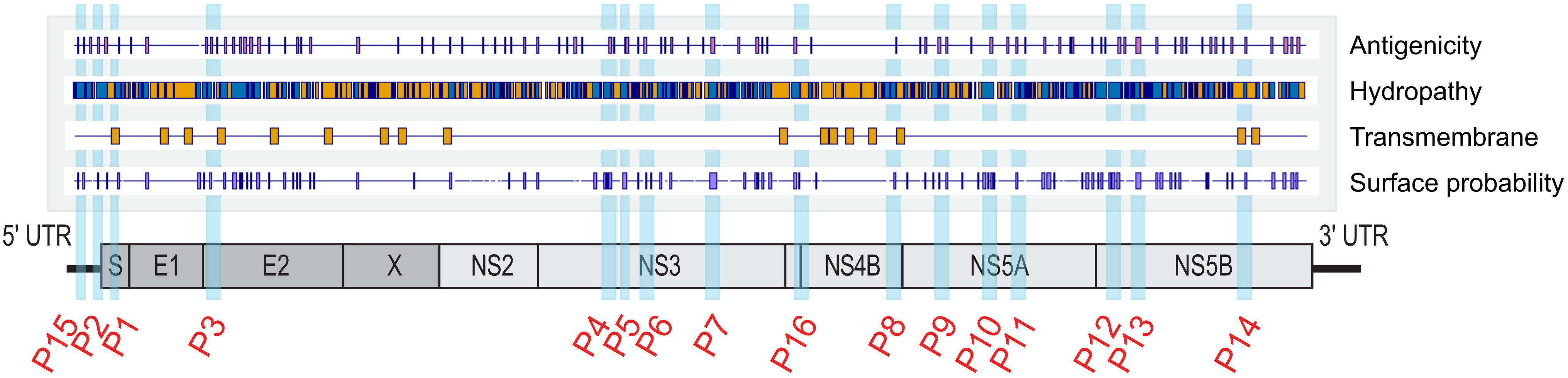

B

Peptide #	Protein	Sequence	% homology with HCV	% homology with GBV-C
1	S	GGSCRSPSRVQVARRVLQLSAFLALIGSGMSSIRSKTEGRIESGQ	8.9%	13.3%
2	S	RDGSLHWSHARHHSVQPDRVAAGPPSVTSVERNMGSSTDQT	17.0%	12.1%
3	E2	SMNSDSPFGTFTRNTESRFSIPRFSPVKINS	9.5%	12.9%
4	NS3	QAPAVTPTYSEITYYAPTGSGKSTKYPVDLVKQGHKVLVL	57.5%	37.5%
5	NS3	VKSMAPYIKETYKIRPEIRAGTGPDGVTVITG	25.0%	31.2%
6	NS3	PETNLRGYAVVISDESHDTSS	42.8%	61.8%
7	NS3	PCTAALRMQRRGRTGRGRRGAYYTTSPGAAPCVS	44.1%	58.8%
8	NS4B	LSERFGQQLSKLSLWRSVYHWAQAREGYTQCG	18.7%	25.0%
9	NS5A	NPTTTGTGTLRPDISDANKLGFRYGVADIVELERRGDKWH	27.5%	15.0%
10	NS5A	QNLAARRRAEYDAWQVRQAVGDEYTRLADEDVD	6.1%	6.1%
11	NS5A	RFVPPVPKPRTRVSGVLERVRMCMRTPPIKF	6.5%	12.9%
12	NS5B	NTTRDHNNGITYTDLVSGRAKP	9.0%	13.6%
13	NS5B	DAPMRIIPKPEVFPRDKSTRKPPRFIVFPGCAARV	34.3%	40.0%
14	NS5B	MPLLCMLIRNEPSQTGTLVT	10.0%	10.0%
15	S + upstream	AEAAPKSGELDSQCDHLAWSFMEGMPTGTLIVQRDGSLH	2.6%	0.0%
16	NS4A-B	SVEVRPAGVTRPDATDETAAYAQRLYQACADSGIFASLQGTASAALGKLA	12.0%	20.0%

S4 Figure. Indices for HPgV-2 polyprotein analysis and peptides for detection of HPgV-2 specific antibody.
