# Supplementary material for: Discovery of a Novel Human Pegivirus in Blood Associated with Hepatitis C Virus Co-Infection
Source: PLoS Pathog. 2015 Dec 11;11(12):e1005325. doi: 10.1371/journal.ppat.1005325 (PMC4676677; doi:10.1371/journal.ppat.1005325)
Supplement: S5 Fig — Multiple amino acid sequence alignment of HPgV-2 peptides 4, 9, and 16 sequences are shown. The number refers to the numerical designation for each of the 10 strains (left column). Shown also are signal to noise cut-off (S/CO) values for each tested peptide. (PDF) [file ppat.1005325.s006.pdf]

Peptide 4 (NS3)

	Sequence	S/CO
125	Q A P A V T P T Y S E I T Y Y A P T G S G K S T K Y P V D L V K Q G H K V L V L	0.9
29	Q A P A V T P T Y S E I T Y Y A P T G S G K S T K Y P V D L V K Q G H K V L V I	-
30	Q A P A V T P T Y S E I T Y Y A P T G S G K S T K Y P V D L V K Q G H K V L V L	-
35	Q A P A V T P T Y S E I T Y Y A P T G S G K S T K Y P V D L V K Q G H K V L V L	-
41	Q A P A V T P T Y S E I T Y Y A P T G S G K S T K Y P V D L V K Q G H K V L V L	-
55	Q A P A V T P T Y S E I T Y Y A P T G S G K S T K Y P V D L V K Q G H K V L V I	-
70	Q A P A V T P T Y S E I T Y Y A P T G S G K S T K Y P V D L V K Q G H K V L V L	-
96		2.3
128	Q A P A V T P T Y S E I T Y Y A P T G S G K S T K Y P V D L V K Q G H K V L V L	-
239	Q A P A V T P T Y S E I T Y Y A P T G S G K S T K Y P V D L V K Q G H K V L V I	-
116	Q A P A V T P T Y S E I T Y Y A P T G S G K S T K Y P V D L V K Q G H K V L V L	-
118	Q A P A V T P T Y S E I T Y Y A P T G S G K S T K Y P V D L V K Q G H K V L V L	-

Peptide 9 (NS5A)

	Sequence	S/CO
125	N P T T T G T G T L R P D I S D A N K L G F R Y G V A D I V E L E R R G D K W H	6.5
29	N P T T T A T G T L R P D I S D A T R L G F R Y G I A E I V E L E L R E H K W H	0.8
30	N P T T T A T G T L R P D I S D A T K L G F R Y G V A E I V E L E W R D N K W H	-
35	N P T T T A T G T L R P D I G D A T R L G F R Y G I A E I V E L E W R G D K W H	-
41	N P T T T A T G T L R P D I G D A T R L G F R Y G I A E I V E L E R R G D K W H	-
55	N P T T T A T G T L R P D I X X A T K L G F R Y G V A D I V E L E X R G D K W H	-
70	N P T T T A T G T L R P D I S D A T K L G F R Y G V A E I V E L E R R G N K W H	3.5
96	N P T T P A T G T L R X D I S D D S K L G F L Y C V A D I V D L E . R R D K W H	6.0
128	N P T T T A T G T L R P D I S D A T K L G F R Y G V A D I V E L E W R G D K W H	4.6
239	N P T T T A T G N L R P D I S D A T K L G F R Y G I A E I V E L E R R G D K W H	-
116	N P T T T A T G T L R P D I S D A T K L G F R Y G V A E I V E L E R R D N K W H	25.5
118	N P T T T A T G T L R P D I S D A T K L G F R Y G V A E I V E L E R R G D K W H	-

Peptide 16 (NS4A-B junction)

	Sequence	S/CO
125	S V E V R P A G V T R P D A T D E T A A Y A Q R L Y Q A C A D S G I F A S L Q G T A S A A L G K L A	13.0
29	S V D N G V A G V T R P D A T D E T A A Y A Q R L Y Q A C A D S G L L A S L Q G T A S A A L S K L A	1.9
30	S V D N G P A G V T R P D A T D E T A A Y A Q R L Y Q A C A D S G I L A S L Q G T A C A A L S K L A	-
35	S V D N G P T G V T R P D A T D E T V A Y A Q R L Y H A C A D S G I L A S L Q G T A C A A L S K L A	-
41	S V D N G P T G V T R P D A T D E T V A Y A Q R L Y H A C A D S G I L A S L Q G T A C A A L S K L A	-
55		1.0
70	S V E N G L A G V T R P D A T D E T A A Y A Q R L Y Q A C A D S G I L A S L Q G T A S A A L S R L A	0.7
96		13.8
128	S V D N G L A G V T R P D A T D E T A A Y A Q R L Y Q A C A D S G I F A S L Q G A A S A A L S R L A	1.6
239	S V D N G L A G V T R P D A T D E T A A Y A Q R L Y Q A C A D S G L F A S L Q G T A S A A L G K L A	-
116	S V E N G L A G V T R P D A T D E T A A Y A Q R L Y Q A C A D S G I L A S L Q G T A S A A L S R L A	26.7
118	S V E N G P A G V T R P H A T D E T A A Y A Q R L Y Q A C A D S G I L A S L Q G T A C A A L S R L A	24.8

Supplementary Figure 5. Alignments of HPgV-2 peptides used in the serological assay
