# Supplementary material for: Discovery of a Novel Human Pegivirus in Blood Associated with Hepatitis C Virus Co-Infection
Source: PLoS Pathog. 2015 Dec 11;11(12):e1005325. doi: 10.1371/journal.ppat.1005325 (PMC4676677; doi:10.1371/journal.ppat.1005325)
Supplement: S3 Fig — The phylogenetic tree was constructed using 8 HPgV-2 strains, representative hepaciviruses, and all fully sequenced pegiviruses in the NCBI GenBank database, including the simian pegiviruses (triangle). Each tree is rooted with yellow fever virus (YFV) as an outgroup. (PDF) [file ppat.1005325.s004.pdf]

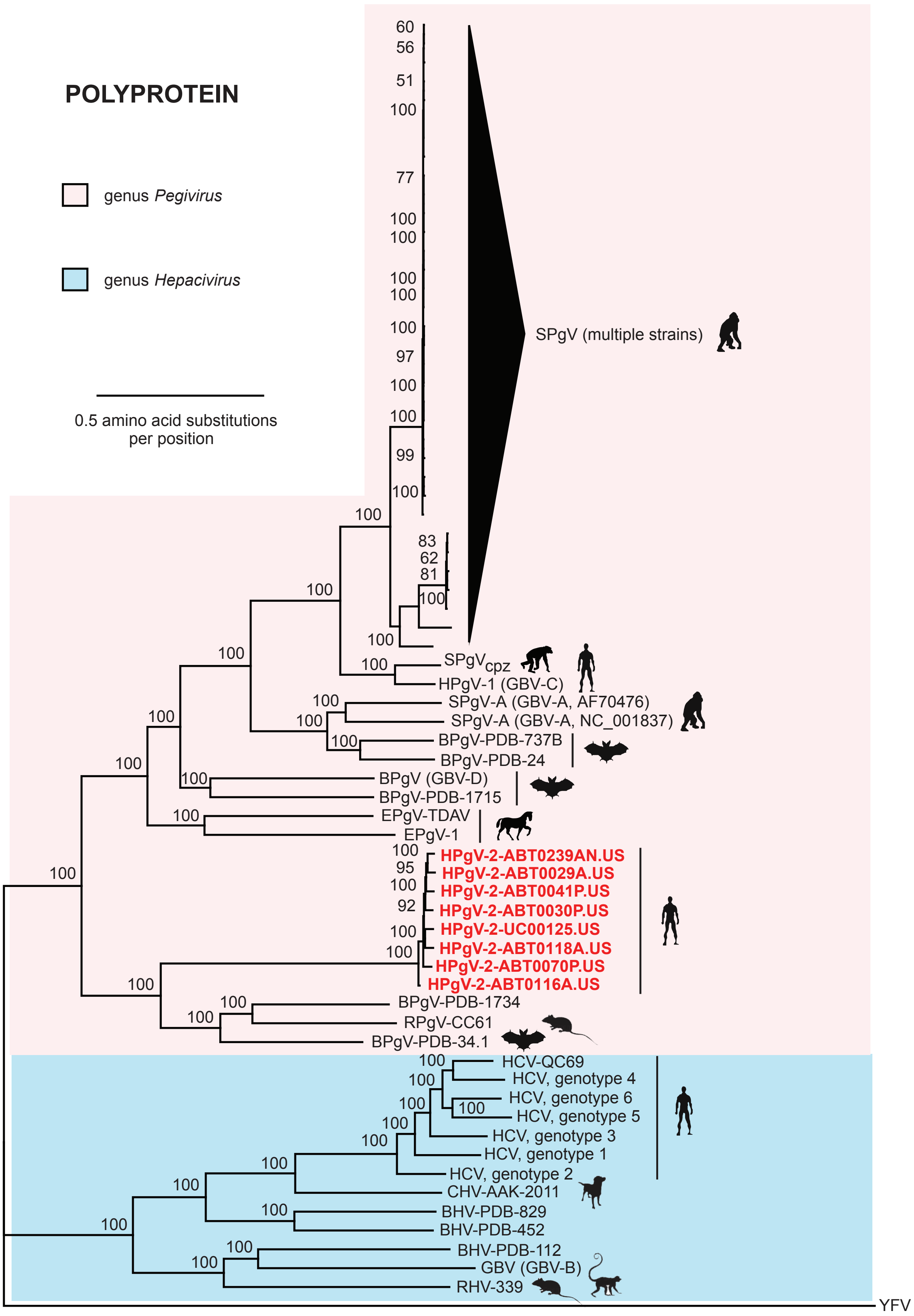

S3 Figure. Phylogenetic analysis of the HPgV-2 polyprotein relative to the polyprotein from other pegiviruses and hepaciviruses
